# Reduced neonatal mortality in a regional hospital in Mozambique linked to a Quality Improvement intervention

**DOI:** 10.1186/s12884-016-1170-y

**Published:** 2016-11-22

**Authors:** Maria Elena Cavicchiolo, Paolo Lanzoni, Mazungo Olivier Wingi, Damiano Pizzol, Marco Daverio, Liviana Da Dalt, Giovanni Putoto, Daniele Trevisanuto

**Affiliations:** 1 Doctors with Africa CUAMM, Padova, Italy; 2 Central Hospital of Beira, Beira, Mozambique; 3 Department of Woman’s and Child’s Health, University of Padova, Via Giustiniani 3, Padova, 35128 Italy

**Keywords:** Neonatal mortality, Low resource setting, Quality improvement, NGO

## Abstract

**Background:**

Neonatal mortality remains a serious health issue especially in low resource countries, where 99% of neonatal deaths occur. Doctors with Africa CUAMM is an Italian non-governmental organization in the field of healthcare that has been working in Africa since 1955. In Mozambique, at the Central Beira Hospital (CBH), it has a project with the aim of supporting the neonatal intensive care unit (NICU) and the Obstetrical Department of the CBH through a multi-level intervention. Our aim was to evaluate the effectiveness of CUAMM continuous Quality Improvement intervention in terms of reduction of the overall neonatal mortality rate in the NICU of CBH.

**Methods:**

A baseline analysis was performed in order to assess the actual standard of neonatal care. Subsequently, the intervention was focused on three main areas: infrastructure, equipment and clinical protocols improvement. A retrospective pre- (2013)/post- (2014) implementation analysis of clinical outcomes was performed.

**Results:**

Total population included 4,276 newborns, 2,118 (50%) born in 2013 and 2158 (50%) born after implementation. Baseline characteristics of the two groups were similar apart from a higher incidence of outborn neonates (33% vs 30%, *p* = 0.02) and a lower incidence of Apgar score < 7 at 5 min (37% vs 43%, *p* < 0.01). The rates of admissions for asphyxia (22% vs 30%), sepsis (4% vs 7%) and prematurity (18% vs 28%) increased between the two study period. Mortality rate for each of these causes decreased from before to after the implementation: asphyxia (34% vs 19%, *p* < 0.01), sepsis (39% vs 28%, *p* = 0.06) and prematurity (43% vs 33%, *p* < 0.01).

**Conclusion:**

We found a reduction in mortality rate among newborns admitted to CBH’s NICU after the first year of CUAMM intervention. Most of this reduction can be attributed to the decrease in deaths for asphyxia, sepsis and prematurity. A Quality Improvement intervention based on infrastructural, equipment and clinical objectives was associated with a reduction of neonatal mortality rate in a low-resource NICU.

## Background

In the last two decades, the global “under-five mortality” has dropped from 90 deaths per 1,000 live births in 1990 to 46 in 2013 [1]. Despite these gains in the under-five mortality, the proportion of deaths that occurs within the first month of life (the neonatal period) has increased from 37% in 1990 to 44% in 2013, showing that declines in neonatal mortality rate is slower than for older children [1, 2]. Neonatal mortality remains a big issue particularly in poor resource countries, where poverty is strongly associated with mortality rate and 99% of the neonatal deaths occurs [2, 3]. Worldwide the main causes of neonatal death are: prematurity (28%), infections (26%), and intrapartum related events (23%) [4].

Mozambique, as the other sub-Saharan African countries, has an extremely high neonatal mortality rate. In 2012, neonatal mortality rate in Mozambique was estimated at 34/1000 live births. The rate of stillbirth was 28/1000 total births. Neonatal deaths constituted 35% of an estimated 85000 deaths under five years of age [4].

Doctors with Africa CUAMM is an Italian non-governmental organization operating in the field of healthcare [5]. It has been working in Africa since 1955 for the promotion of African population’s health. In Mozambique since 1978, CUAMM is now collaborating with the Obstetrical Department and with the Neonatal Intensive Care Unit (NICU) of the Central Beira Hospital (CBH): the second largest city in the country. This project, focused on Child’s and Mother’s Health, started in January 2014 with the aim of supporting the NICU and the Obstetrical Department of the CBH through a multi-level intervention including renovation of the wards, equipment provision and education of the local staff.

The aim of the present study was to assess the effectiveness of CUAMM interventions in the CBH in terms of reduction of the neonatal mortality rate through a retrospective analysis of the pre- and post-implementation data.

## Methods

### Setting and population

The city of Beira has about 500,000 inhabitants, of which 17% have less than 5 years. CBH is a 733-bed government tertiary referring and teaching Hospital for the central region of the Country (population of about 7 million) in Mozambique and the second hospital of the country [6].

At the Obstetrical Department about 5,300 deliveries occur every year (24% of the deliveries expected for the total population), 29% of which are caesarean sections, with a maternal mortality ratio of 520 per 100,000 live births [5]. All the women who delived in the CBH received at least one prenatal visit; no one received antenatal corticosteroids.

The maternal ward is staffed by 13 midwives who have the full responsibility of neonatal management at birth. In all delivery rooms as well as operating rooms, an infant warmer and an emergency neonatal set, including a self-inflating bag, face-masks, suction device and ties, are available.

Criteria for admission to the CBH’s NICU are inborn and outborn patients of all gestational ages up to a postnatal age of 7 days. Before CUAMM intervention, the staff was composed by two nurses, three medical residents and one neonatologist during the morning time till 2.00 p.m. and only one nurse during the rest of the day.

It was a 30-bed unit with about 2,500 admissions per year, 30% of which were outborn [5]. The ward was divided into 3 rooms: one intensive care unit, one for premature babies, and one for post intensive care. Electricity was provided 24 h a day but in case of black out there wasn’t a system of emergency generator. The Kangaroo Mother Care (KMC) was performed in a separated 9-bed room. The NICU was an 8-bed room, provided of a system of non-invasive ventilation (bubble nCPAP), without monitors. Intravenous fluids and first line medications (i.e. antibiotics) were available. The NICU had space heaters to achieve a goal room temperature of 25 °C. A water purification system was not available.

### Baseline survey and interventions

When CUAMM started its support in the CBH in January 2014, baseline data were collected in order to establish the strengths and the weaknesses of the actual care. A multidisciplinary working group composed by the local staff and the Chief of the Pediatric Department identified the strategies for the improvement of the standard of care in the Hospital’s NICU.

The interventions were focused on improving three orders of areas: infrastructure, equipment and clinical protocols. Comprehensive assessments of the quality of care follow the classical Donabedian approach encompassing measures of structure, process and outcome was used (Table 1) [7].

**Table 1:** Quality Improvement interventions classified according to the Donabedian framework

Structures		Process	Outcomes
Structural	Lack of space dedicated to the KMC	Creation of two new rooms with six extra beds for KMC, for a total of fifteen beds	Reduction of neonatal mortality due to asphyxia, prematurity and sepsis
	No bathroom for the mothers	Rehabilitation of one bathroom for the patients and one for the NICU staff	
	No mosquito nets on beds	Painting walls, repairing windows and installation of impregnated mosquito nets for each KMC bed	
Equipment	Lack of equipment and supplies	Oxygen concentrators with implementation of pulse oximeter utilization and technical maintenance	
		Glucometer and point-of-care testing for C-Reactive Protein and technical maintenance	
		UVB light for phototherapy and technical maintenance	
		Infusion pumps and technical maintenance	
		First and second line antibiotics	
Clinical	Lack of standardization of the quality of work between different doctors	Creation of diagnostic-therapeutic protocols for the main neonatal diseases (sepsis, dyspnea, prematurity, asphyxia, hypoglycemia, neonatal seizures, enteral feeding)	
	Lack of internal organization	Monthly meeting with doctors working in the NICU focused on good clinical practice, discussion of case reports, medical issues and protocols	
		Scheduled visiting hours, ward access permitted only to internal personnel and families	
		Organized staff shifts	
		Payment of an extra shift performed by a nurse during the afternoon and night to obtain a nurse/patient ratio of 1/15 compared to 1/30 of the pre-intervention	
	Lack of basic hygienic rules	Meeting twice a month with nurses and NICU’s staff on hand washing, cup feeding, newborn cleaning, water purification for hand washing and formula milk preparation	
	Lack of theoretical knowledge and technical skills about the neonatal resuscitation in the delivery room	Organization of a neonatal resuscitation course for the midwives on January 2014 with a Portuguese-speaking certified neonatologist midwives	
		On-the-job training by a certified local midwife a week per month	
		Weekly meeting with the midwives with discussion of one clinical case	
		Installation of a camera in the delivery room and in the operating room to record 24/7 the neonatal resuscitation made by the midwives working in the Obstetrical Department to evaluate their performances	

The study compared patients admitted to NICU in the pre-intervention phase (1^st^ Jan – 31^th^ Dec, 2013) to the newborns admitted during the first year of CUAMM presence (1^st^ Jan – 31^th^ Dec, 2014).

### Definitions

Prematurity: a newborn weight less than 2,500 g and a maternal fundal height less than 34 cm at the moment of the delivery;

Asphyxia: it was a clinical diagnosis based on the 5 min Apgar score lower than 7 and/or Sarnat & Sarnat score ≥2 at NICU admission; [8]

Sepsis: it was a clinical diagnosis based on neurologic examination, detection of moderate hypothermia (<36 ° C) or hyperthermia (>37.5 ° C), respiratory distress (tachypnea, chest wall retractions, expiratory grunting, nasal flaring and cyanosis) and breastfeeding difficulties;

Low birth weight (LBW): patients with a birth weight less than 2,500 g at birth;

Very low birth weight (VLBW): patients with a birth weight less than 1,500 g at birth.

### Data collection

Data extraction was performed by a trained external member (not involved in clinical activity nor in the study design) by using a piloted clinical report form in order to ensure consistent data collection. Chart abstraction was performed in accordance with previously published guidelines [9]. The abstractor was not blinded to the study question. Data were collected by reviewing all the original medical records as well as the clinical electronic databases.

### Statistical analysis

Descriptive statistics were used to compare baseline characteristics of the study groups. A bivariate analysis was performed to evaluate the differences observed between the pre- and post- intervention population. For binary variables the *chi-square test* was used. A *p*-value < 0.05 was considered statistically significant. All data were analysed with SPSS 17.0 for Windows (IBM SPSS Statistics, IBM Corporation, Chicago, IL).

## Results

During the study period, 4,276 patients were admitted to the NICU, 2,118 (50%) in the pre-intervention phase and 2,158 (50%) during the first year of CUAMM presence.

The baseline characteristics of the two groups were similar apart from incidence of outborn neonates (30% vs 33%, *p* = 0.02), incidence of Apgar score < 7 at 5 min (43% vs 37%, *p* < 0.01), and number of VLBW (5% vs. 4%, *p* = 0.02) (Table 2).

**Table 2:** Demographic and clinical characteristics of patients admitted to the NICU pre- versus post- CUAMM interventions

	Pre-intervention *n* = 2118	Post-intervention *n* = 2158	*p*
Male gender, n (%)	1102 (52)	1187 (55)	0.05
Outborn babies, n (%)	635 (30)	715 (33)	0.02
Caesarean sections, n (%)	614 (29)	627 (29)	0.96
5-min Apgar score < 7, n (%)	911 (43)	801 (37)	<0.01
Birth weight 1,500–2,499 g, n (%)	349 (16)	357 (17)	0.95
Birth weight 1,000–1,499 g, n (%)	104 (5)	77 (4)	0.02

Table 3 summarizes the admissions and the deaths in relation to the diagnosis of the patients during the two study periods. Overall neonatal mortality rate decreased from 26 to 18% (*p* < 0.01) after the CUAMM intervention; no differences were noted in mortality rate of outborn infants during the 2 study periods. Despite the significant higher rate of admissions for asphyxia (22% vs 30%), sepsis (4% vs 7%) and prematurity (18% vs 28%), the mortality rate for each of these causes decreased: asphyxia (34% vs 19%, *p* < 0.01), sepsis (39% vs 28%, *p* = 0.06) and prematurity (43% vs 33%, *p* < 0.01).

**Table 3:** Admissions and deaths in relation to the diagnosis of all patients admitted to the NICU (pre- versus post- CUAMM intervention)

	Pre-intervention *n* = 2118	Post-intervention *n* = 2158	*p*
N° of deaths, n (%)			
Total	544 (26)	396 (18)	<0.01
Outborn patients	274 (50)	199 (50)	0.97
Admission for asphyxia, n (%)	492 (20)	641 (30)	<0.01
Deaths for asphyxia, n (%)	168 (34)	122 (19)	<0.01
Admission for sepsis, n (%)	111 (4)	158 (7)	<0.01
Deaths for sepsis, n (%)	43 (39)	44 (28)	0.06
Admission for prematurity, n (%)	447 (18)	605 (28)	<0.01
Deaths for prematurity, n (%)	192 (43)	200 (33)	<0.01

## Discussion

This study shows a reduction in mortality rate among infants admitted in the CBH’s NICU after CUAMM intervention. Most of this success can be attributed to prematurity and asphyxia reduction.

Preterm infants are rising globally, both in high as well as low income countries [4]. A complex approach including thermal control, respiratory support, infection prevention and optimization of fluid/caloric intake is indicated in these vulnerable patients. Besides to these interventions, we implemented the KMC approach at the CBH.

Improved overall survival of premature infants in poor resource settings is, among the others, due to the introduction of KMC approach [10]. The KMC is an evidence-based approach that has been demonstrated to reduce mortality and morbidity in preterm infants in poor resources countries. Several meta-analysis show that KMC significantly reduces preterm mortality and improves other outcomes including sepsis, emotional attachment in mothers, and weight gain when compared to conventional care in preterm infants [10–13].

The implementation of this evidence-based method in the CBH's NICU could explain, at least partly, the reduction in neonatal mortality rate due to prematurity.

Between 5–10% of all babies born in facilities need some degree of resuscitation, such as tactile stimulation or airway clearing or positioning and approximately 3–6% require basic neonatal resuscitation, consisting of simple initial steps and assisted ventilation [14, 15]. These procedures can reduce intrapartum-related neonatal deaths by 30% [16]. In many developing countries, an inability to offer effective newborn resuscitation has been tolerated for many years, reflecting the belief that resuscitation is complex and dependent on the presence of expensive technology impossible to apply in low-income health systems [14]. In 2006, Newton and English wrote a systematic review demonstrating that is possible to provide resuscitation with simple equipment and minimal skills, without compromising the quality of the intervention [17]. In the last decade in more than 70 poor incoming countries newborn resuscitation programs, designed to teach basic knowledge and skill in under resources settings, have been performed with good results in terms of reduction of mortality and morbidity for neonatal asphyxia [18–20].

Based on this evidence, in January 2014, CUAMM organized a neonatal resuscitation course to the midwives of the CBH. Before and after the course the intervention on the newborn needing resuscitation were video recording and analyzed by an expert neonatologist in Italy. These videos showed an improvement on the quality of the resuscitations [21]. In addition to the course, the educational program included the training of a local midwife responsible of a continuous, weekly “on the job training” to the colleagues of the Obstetrical Department. Taken together, these educational initiatives could explain the decreased mortality rate due to asphyxia. We consider to continue supporting this program on resuscitative maneuvers because previous work showed a decay of staff performance over time [22].

Nevertheless, the number of patients admitted for asphyxia (492 vs 641, *p* < 0.01) increased after CUAMM intervention. These findings could have different explanations: the effect of educational intervention on assigning Apgar score, the increased number of admissions of patients suffered from milder asphyxia and the improved management of these patients during hospital stays.

Despite the training on hand wash, the creation of protocols on neonatal sepsis’s treatment of and antibiotics supply, the mortality rate for sepsis did not decrease as expected. In a hospital survey conducted in the first trimester of 2014, only 78% of the NICU staff washed the hands properly. This number was even lower in the Obstetrical Department (about 30%) [23].

Our program included the provision of a point-of-care reader for C-Reactive Protein (CRP). However, we realized that it was used rarely, due to the lack of confidence and trust of the instrument by the local staff. However, when a clinical diagnosis of sepsis was suspected and CRP was negative, the antibiotic treatment was started. Serial CRP measurements were not used for interrupting antibiotic treatment.

Our study has some limitations due to the retrospective design itself. Misclassification can be occurred particularly in defining LBW infants and prematurity and in attributing causes of death because most of the diagnoses were clinically based, without any laboratory or instrumental support.

Finally, although, our initiatives, taken together, were able to reduce mortality rate in this referral low resource NICU, it was not possible to exactly determine the role of each intervention on the final positive outcome.

Future Quality Improvement interventions for reducing mortality and morbidity rate will focus on implementing the actual program and progressively introducing new strategies such as the disinfection of the umbilical cord with chlorhexidine, introduction of antenatal corticosteroid prophylaxis (actually not provided in Beira) and education on the use of devices for non-invasive respiratory support. Monthly meetings on maternal and neonatal deaths between Obstetrical and Pediatric staff are routinely held at CBH. They are based on the revision of the medical reports, without a well-structured evaluation tool. Specific instruments to guide these discussions and provide useful feedbacks to the staff should be recommended.

As during the study period there was neither a better electricity supplies nor a increasing of the healthcare providers’salaries, these aspects should be taken into account in the future.

The sustainability of this project was initially due to the collaboration between CUAMM and the CBH. Local administration has been planning to financially support these Quality Improvements at the end of the CUAMM project in 2017, suggesting that the collaboration with local political, administrative and clinical staff is a key point to maintain the changes over time.

## Conclusions

Our study shows that a Quality Improvement intervention based on infrastructure, equipment and clinical protocols was associated to a reduction of neonatal mortality rate, especially that due to prematurity and asphyxia in a low-resource setting. Randomized controlled trials are needed to evaluate the real impact of this intervention, excluding confounding factors.

## Abbreviations

CBH: Central Beira Hospital
CRP: C-Reactive Protein
KMC: Kangaroo Mother Care
LBW: low birth weight
NICU: Neonatal Intensive Care Unit
VLBW: very low birth weight
